# The relationship between emotional intelligence, anxiety, and performance in physical education and sport students

**DOI:** 10.3389/fpsyg.2023.1236070

**Published:** 2023-10-12

**Authors:** Wael Zoghlami, Aymen Hawani, Hyem Khiari, Sofiene Mnedla, Santo Marsigliante, Ali Elloumi, Antonella Muscella

**Affiliations:** ^1^Higher Institute of Sport and Physical Education, Sfax, Tunisia; ^2^Higher Institute of Sport and Physical Education Ksar-Said, Tunis, Tunisia; ^3^Physical Activity, Sport and Health, Research Unit (UR18JS01), National Observatory of Sport, Tunis, Tunisia; ^4^Faculty of Medicine of Tunis, Tunis, Tunisia; ^5^Higher Institute of Humanities, Zaghouan, Tunisia; ^6^Department of Arts and Social Sciences, Sfax University, Sfax, Tunisia

**Keywords:** emotional intelligence, anxiety, sport, performance, physical education

## Abstract

Emotional intelligence (EI) has been recently the main target in research on sports psychology. The objectives of this study were to investigate the relation between self-report measures of emotional intelligence, pre-competitive anxiety, and performance among students during the physical education exam of the high school final year. This cross-sectional study was conducted on a sample of 350 students attending the physical education exam in the year 2021–2022. Analysis of the correlations between the scores of Emotional intelligence and anxiety showed that self-confidence was positively correlated with all dimensions of Emotional intelligence (Beta = 0.524; *p* = 0.000). Multivariate linear regression analysis showed that the most related items to performance were self-confidence in a positive direction (*r* = 0.56; *p* = 0.000) and negatively with somatic anxiety (*r* = −0, 39; *p* = 0.000). Considering the Emotional intelligence subscales, hetero-emotional management was significantly positively associated with performance (*p* = 0.000) however emotional perception was negatively correlated with performance (*p* = 0.003). These results demonstrate the importance of social and emotional learning programs for improving self-confidence and better management of emotions during physical education and sports.

## 1. Introduction

Emotions have always been the main target of research in psychology (Izard, 2009). Knowledge of the causes and the consequences of emotions as well as the management of emotional situations constitute important elements to achieving high-performance (Tyng et al., 2017). Many contemporary conceptual orientations have taken a positive psychology perspective regarding the identification of performance-facilitating influences such as emotional intelligence (Sánchez-Álvarez et al., 2020). The concept of emotional intelligence (EI) has been defined since 1997 by Salovey and Sluyter as the ability of the person to identify and understand his emotions and those of others and to regulate them to achieve optimal performance (Salovey and Sluyter, 1997). Anxiety is also one of the most studied emotional aspects in psychology. According to the literature, performance can be negatively affected by stressing factors mainly during competitions and exams (Sanhueza et al., 2016). Anxiety has been defined as negative feelings and tension resulting from environmental demands associated with arousal (Rowland and van Lankveld, 2019). These demands are usually stressful, indicating a perceived imbalance between the given demand and their ability to meet the demand (Ford et al., 2017).

Interest in EI has spawned work in multiple fields, including sports and physical education, which is particularly fertile ground to explore due to the preponderance of emotions with an objective of high-performance (Laborde et al., 2013; Kopp and Jekauc, 2018). EI plays an important role in physiological responses to stress and anxiety, and their results on performance (Lea et al., 2019). In sports, managing emotions and anxiety is considered one of the keys to performance (Tur-Porcar and Ribeiro-Soriano, 2020). So, according to the literature, there is a relationship between emotions, EI, and sports performance (Meyer and Fletcher, 2007; Lane et al., 2012; Üngür and Karagözoğlu, 2013). Thus, the meaning of this relationship remains controversial. The meta-analysis of Laborde et al. (2016) on EI summarized the results of six studies examining the relationship between EI and athletic performance and concluded with conflicting results and suggested the need for further studies. In the Arabic world and Tunisia data on this subject are scarce. In this order, this study was conducted to study the relation between self-reported measures of emotional intelligence, anxiety, and performance among students during the physical education exam of the final year of high school, constituting a real stressful situation with an objective of high performance. Thus, we hypothesized that being the EI and its dimensions negatively correlated with anxiety, dimensions of anxiety are negatively correlated to high student performance, while EI is positively correlated. The aim of this study was to investigate the impact of EI and anxiety on students’ performance during the final high school physical education exam, with the aim of revealing this, presumably negative, relationship between EI and physical performance.

## 2. Methods

### 2.1. Participants

This cross-sectional study was conducted on a sample of 350 students attending the physical education exam of the final year high school governorate of Zaghouan (Center west of Tunisia).

We used an online program^1^ to estimate the minimal sample size needed in this study. Multi-level sampling was used to determine the needed sample for this study. Schools and classes were determined by drawing lots.

In detail, we have chosen: 4 schools in the Governorate of Zaghouan using simple random sampling, 5 classes within each school using systematic sampling method, and 20 students from each school using simple random method. We used a Random Number Generator program to create a list of random numbers, based on our specifications. The students were selected according to an identifier accorded to each, obtaining the list of a representative sample of students.

Students in the high-year physical education exam were included in the study; they were healthy and free of any disabilities, musculoskeletal, neurological, or respiratory diseases or dysfunctions, and without any history of mental disorders or psychiatric drugs. Exclusion criteria were incomplete questionnaires. The anthropometric characteristics of students (mean ± SD) are shown in Table 1.

**Table 1:** Clinical characteristics of the participant.

Characteristics	Mean ± S.D.
Age (year)	18.77 ± 0.76
Height (cm)	170.94 ± 9.73
Weight (kg)	64.30 ± 9.98.
Body mass index (kg/m^2^)	22.47 ± 9.71

S.D, standard deviation.

After approval of the concerned comities, the study investigators (four physical education professors) were present during the final high school physical education exam from the 18 to the 27 April, of the 2021/2022 school year, 8 a.m. to 11:30 a.m. to fill the questionnaires just before the exam. The investigators asked for the approval of each student to participate in the study (from the selected sample of students) and explained how to complete the questionnaires; then, asked them to complete the two questionnaires (SSRI and CSAI-2r). Each questionnaire took from 10 to 15 min to be completed.

The study was conducted according to the latest version of the Declaration of Helsinki, and the protocol was fully approved by the Local Ethics Committee of the National Center of Medicine and Science of Sports of Tunis (CNMSS-LR09SEP01) before the commencement of the procedure.

To estimate the minimal sample size needed in this study, we used the following formula:


$$n=\frac{{\mathrm{NZ}}^{2}\mathrm{pq}}{\left(e^{2}\left(N\_1\right)+Z^{2}\mathrm{pq}\right)}$$

where n = sample size; N = population size; Z = the statistic corresponding to level of confidence; p = probability of success; q = probability of failure; e = confidence interval. With a confidence level of 95%, a confidence interval of 5%, and the probability of success at 50%, the sample size required (depending on the Universe) was around 385.

### 2.2. Study measures

Two questionnaires were used to study the emotional intelligence and anxiety state among the studied population.

- The Schutte self-report emotional intelligence test: the Schutte Self-Report Inventory (SSRI) was used to assess the as previously described by Zoghlami et al. (2022) and Schutte et al. (1998). EI was measured using the Schutte Self-Report Inventory also called Schutte Self-Report Emotional Intelligence Test. This scale measures the participants’ perception of their emotional skills at an intrapersonal and interpersonal level. It consists of 33 Likert items answered on a five-point scale (1 = strongly disagree, 2 = disagree, 3 = neutral, 4 = agree, and 5 = strongly agree). The EI scale describes four factors as follows: perception of emotions, managing self-emotions, managing others’ emotions, and utilizing emotions (emotional use). We used a validated (but still unpublished) Arabic version of the scale which showed good psychometric properties.
- The revised inventory-2 of the anxiety state of the competition (Martinent et al., 2010): This version of the questionnaire contains 17 items and evaluates three main dimensions: cognitive anxiety, somatic anxiety, and self-confidence (Cox et al., 2003). Cognitive anxiety involves cognitions about possible failure, while somatic anxiety involves the perception of bodily symptoms and heightened negative arousal. Self-confidence, on the other hand, involves cognition that one is up to the task and able to give one’s best possible performance. We used a validated Arabic-Tunisian version (Hajji and Elloumi, 2017).
- The Performance in this study was based on the result of the final physical education high school exam (over 20). The final high school exam results were considered as ‘Good ‘or ‘poor to medium’. This method facilitated the regression analysis, which needed a binary qualitative variable.

### 2.3. Procedure

Several investigators (physical education professors) were present (after approval of the concerned comities) during the final high school exam of physical education in the governorate of Zaghouan. Each student was given to complete the two questionnaires (SSRI and CSAI-2r) just before passing the exam. Investigators obtained the approval of the participant and explained how to complete the questionnaires.

### 2.4. Data analysis

Data were analyzed with Statistical Package for Social Sciences (SPSS) software, version 17. Descriptive data are presented as percentages or as means and Standard Deviations (SDs). We tested the normality of all the continuous variables using the test of Kolmogrov-Smirnov [a variable is normally distributed if the Kolmogrov test is not significant (*p* > 0.05)]. Simple linear regression was used to explore relations between scores of emotional intelligence scales, anxiety subscales (SA, CA, and SC), and performance. Then multiple regression was performed to study the relation between anxiety, EI adjusted subscales, and performance. The limit of significance was fixed at 0.05.

### 2.5. Ethical considerations

We have obtained approval from the Youth and Sports Commission of the governorate of Zaghouan. Investigators obtained the participant’s approval before the interview and explained the study’s procedure and purpose.

## 3. Results

**Figure 1:**
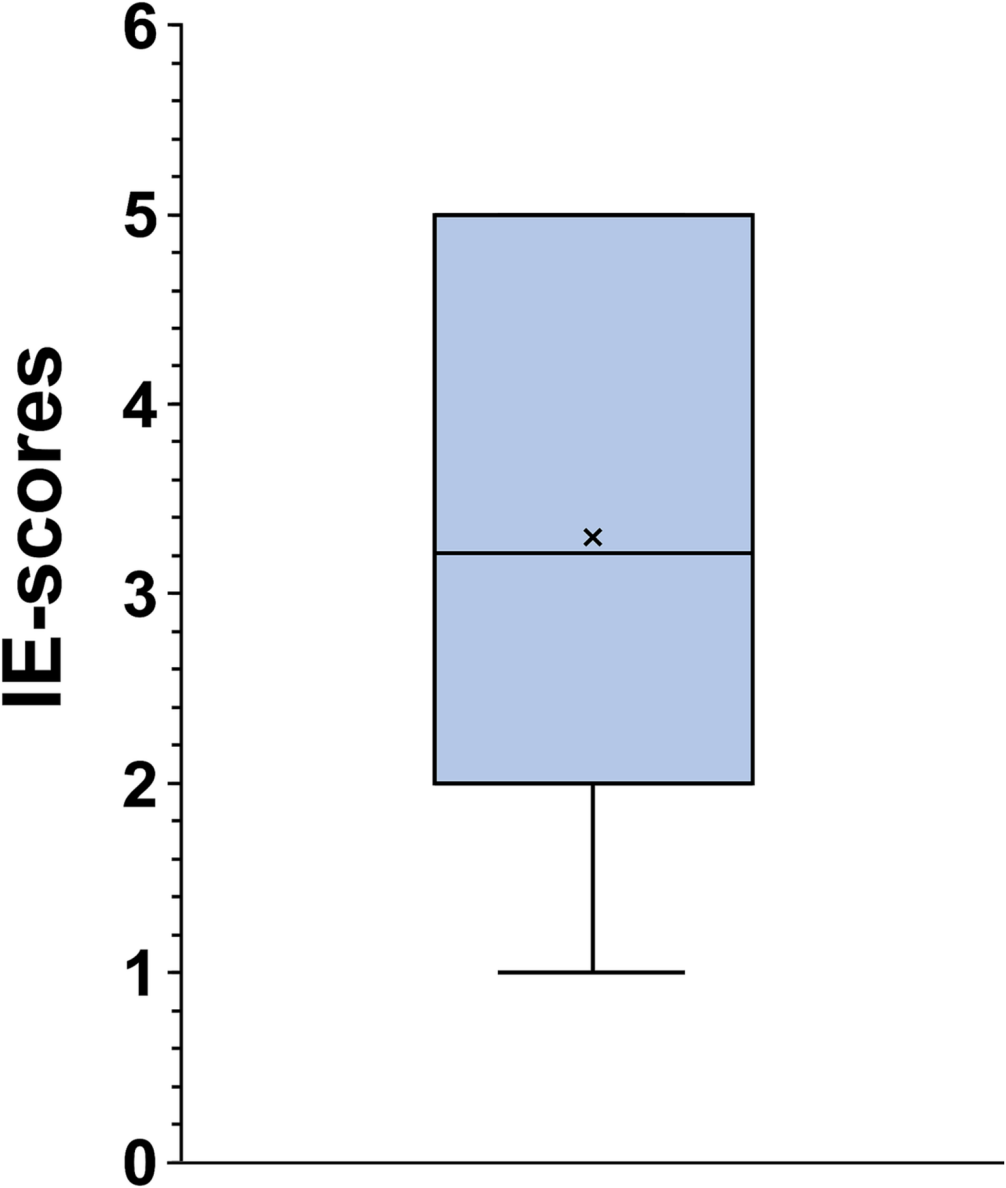
Scores of the items for emotional intelligence (EI). In this representation, the central box covers the middle 50% of the data values, between the upper and lower quartiles. The bars extend out to the extremes, while the central line is at the median.

The present study was about 350 high school students during the final year physical education exam. The mean age of the studied population was 18.7 year ±0.76 and the gender ratio was 0.96 (49% males). The mean performance score among the studied population was 14.6 ± 3.9. The average EI score was 113 ± 20.8, the median was 112 with extremes ranging from 60 to 150. The 25th and the 75th quartiles were 96 and 133. Considering the different dimensions, the use of emotions had the highest average score of 3.7 ± 0.9. The mean scores for managing own and others’ emotions were 3.6 ± 0.8 and 3.4 ± 0.7. Perception of emotions had the lowest score of 3.2 ± 0.6 (Figure 1).

**Figure 2:**
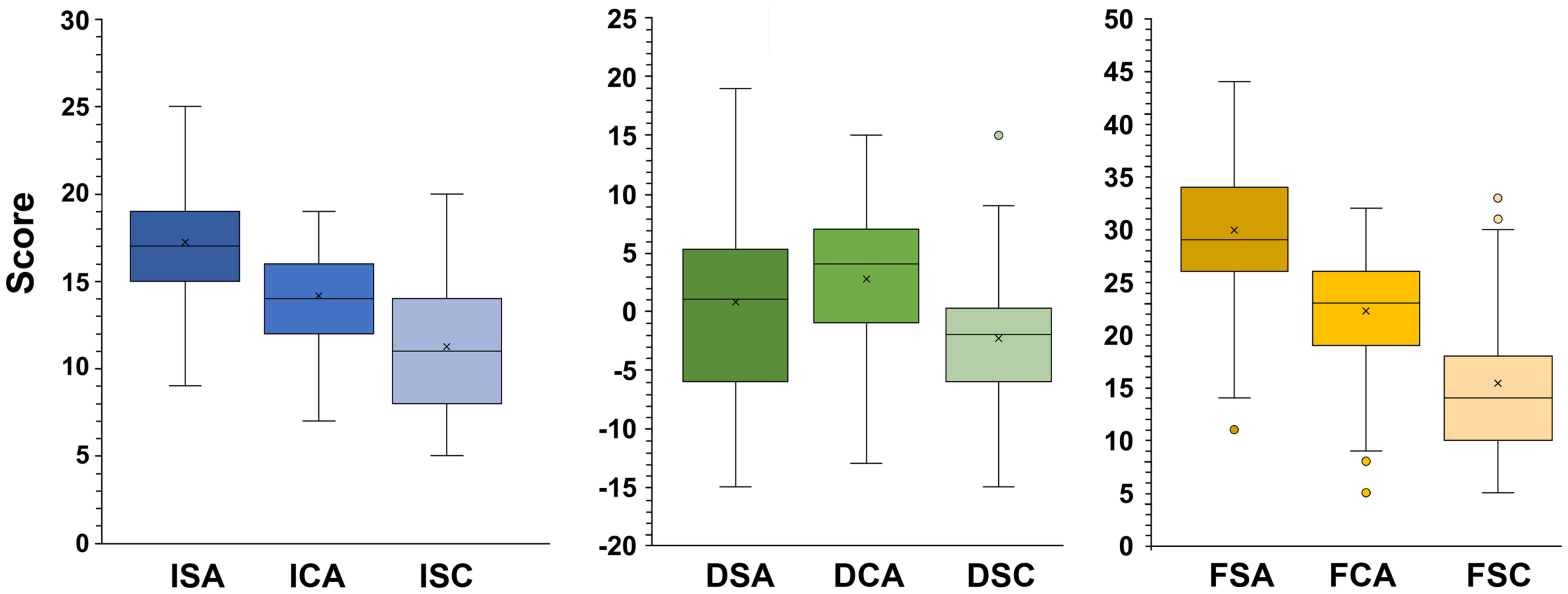
Score distribution for each subscale of the competition competitive state anxiety inventory. In this representation, the central box covers the middle 50% of the data values, between the upper and lower quartiles. Those values that are beyond 1.5 times the interquartile range beyond the central box are plotted as individual points. The bars extend out to the extremes, while the central line is at the median. ISA, Intensity Somatic Anxiety; ICA, Intensity Cognitive Anxiety; ISC, Intensity Self-Confidence; DSA, Direction Somatic Anxiety; DCA, Direction Cognitive Anxiety; DSC, Direction Self-Confidence; FSA, Frequency Somatic Anxiety; FCA, Frequency Cognitive Anxiety; FSC, Frequency Self-Confidence.

Regarding anxiety, the highest average score in terms of intensity and frequency was that of somatic anxiety, followed by the score of cognitive anxiety, and finally, self-confidence (Figure 2).

The mean of results corresponding to students’ performance was equal to the median of 15 ± 3.7, with extremes varying from 4 to 20.

### 3.1. Association between emotional intelligence, anxiety, and performance

We first tested the normality of all continuous variables, and the Kolmogorov test was not significant (*p* > 0.05). When considering the different dimensions, the most important association was observed between auto-emotional management and performance (Beta = 0.419; *p* = 0.000; Table 2). The average anxiety score among the studied population was 2.15 ± 0.44. Regarding anxiety subscales: Self-confidence was the most significantly associated item with the performance in a positive direction (Beta = +0.524; *p* = 0.000). There was also a significant association between somatic anxiety and performance in a negative direction (Beta = −0.333; *p* = 0.000; Table 2).

**Table 2:** Univariate analysis between EI, anxiety, and performance.

		Bêta	T	P
EI	Emotional perception	−0.285	−2.874	0.004
	Auto-emotional management	0.419	4.732	0.000
	Hetero-emotional management	0.148	1.801	0.073
	Emotional use	0.226	2.023	0.044
Anxiety	Somatic anxiety	−0.333	−5.610	0.000
	Cognitive anxiety	0.016	0.270	0.788
	Self confidence	0.524	10.526	0.000

### 3.2. Correlations between anxiety items and performance

Correlation coefficients between dimensions of anxiety and performance were very significant in a positive direction for self-confidence (*r* = 0.56; *p* = 0.000) and in a negative direction for somatic anxiety (*r* = −0.39; *p* = 0.000; Table 3).

**Table 3:** Correlation coefficients between dimensions of anxiety and performance.

	Somatic anxiety	Cogntive anxiety	Self confidence	Anxiety State	Performance
Somatic anxiety	1				
Cognitive anxiety	0.547^**^	1			
*P* value	0.000				
Self confidence	−0.125	−0.055	1		
*P* value	0.054	0.398			
Anxiety State	0.592^**^	0.687^**^	0.607^**^	1	
*P* value	0.000	0.000	0.000		
Performance	−0.390^**^	−0.195^**^	0.564^**^	0.132^*^	1
*P* value	0.000	0.002	0.000	0.041	

*p<0.05; **p<0.001.

### 3.3. Correlations between emotional intelligence items and performance

Correlation coefficients between dimensions of EI and performance were very significant in a positive direction with coefficients of 0.48 for auto-emotional management, 0.42 for emotional use, and 0.36 for Hetero-emotional management. The total EI score was correlated positively with performance with *r* = 0.44 (Table 4).

**Table 4:** Correlation coefficients between dimensions of EI and performance.

	Performance	Perception of emotions	Auto-emotional management	Hetero-emotional management	Emotional use	EI
Performance	1					
Perception	0.288^**^	1				
Auto-emotional management	0.484^**^	0.690^**^	1			
Hetero-emotional management	0.361^**^	0.688^**^	0.597^**^	1		
Emotional use	0.422^**^	0.804^**^	0.767^**^	0.703^**^	1	
EI	0.443^**^	0.900^**^	0.876^**^	0.824^**^	0.932^**^	1

**p<0.001.

### 3.4. Correlation between emotional intelligence scales and anxiety

Analysis of the correlation between the dimensions of EI and anxiety showed that self-confidence was positively correlated with all dimensions of EI: the most important correlation was the management of one’s emotions (*r* = 0.501; *p* = 0.01) followed using emotions (*r* = 0.441; *p* = 0.01). somatic anxiety was negatively correlated with all dimensions of EI: essentially the use of emotions (*r* = −0.38; *p* = 0.01) and the management of emotions (*r* = −0.306; *p* = 0.01). Cognitive anxiety was also negatively correlated with the dimensions of EI; the highest correlations were the perception of emotions (*r* = −0.328; *p* = 0.01) and the management of one’s emotions (*r* = −0.321; *p* = 0.01; Table 5).

**Table 5:** Correlation between IE and anxiety dimensions.

	Somatic anxiety	Cognitive anxiety	Self confidence	Perception of emotions	Management of one’s emotions	Management of other’s emotions	Use of emotions
Somatic anxiety	1						
Cognitive anxiety	0.547^**^	1					
Self confidence	−0.125	−0.055	1				
Perception of emotions	−0.224^**^	−0.328^**^	0.397^**^	1			
Management of one’s emotions	−0.306^**^	−0.321^**^	0.501^**^	0.690^**^	1		
Management of other’s emotions	−0.177^**^	−0.138^*^	0.274^**^	0.688^**^	0.597^**^	1	
Use of emotions	−0.388^**^	−0.279^**^	0.441^**^	0.804^**^	0.767^**^	0.703^**^	1

*p<0.05; **p<0.001.

### 3.5. Multivariate analysis between anxiety, emotional intelligence, and performance

After multivariate linear regression analysis, self-confidence was related to performance in a positive direction and somatic anxiety in a negative one. Considering EI subscales, Hetero-emotional management had a significant association with performance in a positive direction however, emotional perception was related to performance in a negative direction. In a less important but significant relation, auto-emotional management was also related to performance (Table 6).

**Table 6:** Multivariate linear regression between Anxiety subscales and EI regarding performance.

	Bêta	T	*P*
Somatic anxiety	−0.290	−4.704	0.000
Cognitive anxiety	−0.002	−0.039	0.969
Self confidence	0.470	8.435	0.000
Emotional perception	−0.264	−2.974	0.003
Auto-emotional management	0.207	2.601	0.010
Hetero-emotional management	0.255	3.604	0.000
Emotional use	0.024	0.232	0.817

## 4. Discussion

In this study we hypothesized and demonstrated the existence of a relationship between emotional intelligence, competitive anxiety, and student performance, focusing on it during the final high school physical education exam. According to the results of the present study, self-confidence was significantly related to performance in a positive direction. One of the most consistent findings in the peak performance literature is the significant correlation between self-confidence and successful sporting performance (Smith and Bar-Eli, 2007). Confidence has been consistently associated with positive affect, whereas a lack of confidence has been associated with anxiety, depression, and dissatisfaction (Martens et al., 1990). According to Vealey and Campbell (1988), subsequent levels of sports confidence influence an athlete’s thoughts, feelings, and behaviors, which determine sporting performance. Self-confidence would be directly related to performance whereas there is a complex relationship between anxiety and performance. Anxiety consists of two subcomponents: cognitive and somatic anxiety, which influence performance (Parnabas et al., 2013). According to our findings, somatic anxiety was associated with performance in a negative direction which is consistent with the literature (Mercader-Rubio et al., 2023). However, there was no significant association between cognitive anxiety and performance in this study, as in the previous study (Filippi et al., 2022). The level of competitive anxiety among athletes differs according to individuals (Hanin, 2003). Many types of research showed that winning in a competition depends on how athletes can control their anxiety levels. The athlete needs an optimal level of anxiety to perform well (Wagstaff, 2014; Martinent et al., 2015; Kopp and Jekauc, 2018). According to the literature, athletes’ ability to regulate individuals’ emotions involved in sporting performance is necessary to control and influence social activity to succeed at the highest level of competition in peak performance in competitive sports (Lane et al., 2009; Arribas-Galarraga et al., 2020; Tamminen et al., 2021). A meta-analysis investigation by Kopp and Jekauc (2018) concluded that a person high in El is good at recognizing and regulating emotions of self and others, can enhance positive emotion, and channel away negative emotions in self and others (Zoghlami et al., 2022). This was consistent with the findings of the present study which showed a significant positive association between auto and hetero-emotional management regarding performance whereas emotional perception was significantly associated with performance in a negative direction. Thus, the challenge behind perceptions as reality is not only to be aware of them but also to be able to transform negative perceptions into more positive ones. In fact, at the individual level, higher EI was found to be positively related to the use of psychological skills, such as imagery and self-talk (Lane et al., 2009) which have been proven to aid performance. Another positive effect of EI in sports is its effect on stress when under pressure (Arribas-Galarraga et al., 2020).

According to Kopp and Jekauc (2018), practitioners, such as applied sports psychologists, coaching staff, athletes, and sports administrators, need appropriate knowledge of the role of EI and its relevance for successful performance in major competitions. They also should promote the implementation of EI screening and EI development programs as an integral part of the training process.

In summary, although it is known that anxiety and EI are differently correlated with academic performance, this is the first study to show relationships with physical performance, in a group of students, during the final physical exam of high school.

These relationships have also been studied in young professional athletes (Castro-Sánchez et al., 2019), however, all our results are novel because they show in detail the effects of the dimensions of anxiety and EI on physical performance in students.

In particular, somatic anxiety is negatively correlated to high students’ performance; but such physical performance was not significantly associated with cognitive anxiety.

In our participants, EI was negatively correlated with somatic and cognitive anxiety. In addition, this study has shown how much high emotional Intelligence (especially management of emotions), self-confidence, and the management of anxiety are important to raise the performance among students in physical education and sports learning.

Nevertheless, this study contains some limitations such as the sample size which could have been larger, also other more detailed questionnaires to explore emotional intelligence could have been used but the conditions of the final high school physical education exam did not allow for an increase in the number of sizes of the population and the use of longer questionnaires. This study was about students, so the results are specific to this population. Further studies to explore the relationship between EI, anxiety, and performance among athletes of different specialties are recommended.

## 5. Conclusion

The findings of the present study showed that the most related item to performance among students during the final physical exam of high school was self-confidence in a positive direction. Emotional perception was independently and negatively associated with performance however hetero-emotional management was significantly associated with performance in a positive direction. These results lead to the importance of social and emotional learning programs to improve self-confidence and better management of emotions in Physical education and sports learning. Further studies are recommended to explore the interaction between EI and sports performance.

## Data availability statement

The raw data supporting the conclusions of this article will be made available by the authors, without undue reservation.

## Ethics statement

The studies involving humans were approved by Local Ethics Committee of the National Center of Medicine and Science of Sports of Tunis (CNMSS-LR09SEP01). The studies were conducted in accordance with the local legislation and institutional requirements. The participants provided their written informed consent to participate in this study.

## Author contributions

WZ, AH, AE, and AM contributed to conception and design of the study. WZ and AH organized the database. WZ and KH performed the statistical analysis. WZ wrote the first draft of the manuscript. WZ, SMn, AM, and SMa wrote sections of the manuscript. All authors contributed to the article and approved the submitted version.

## Footnotes

^1^ https://www.openepi.com/SampleSize/SSPropor.htm

## References

[ref1] Arribas-Galarraga S. Cecchini J. A. Luis-De-Cos I. Saies E. Luis-De Cos G. (2020). Influence of emotional intelligence on sport performance in elite canoeist. J. Human Sport Exerc. 15, 772–782. doi: 10.14198/jhse.2020.154.05

[ref2] Castro-Sánchez M. Zurita-Ortega F. Ubago-Jiménez J. L. González-Valero G. Chacón-Cuberos R. (2019). Relationships between anxiety, emotional intelligence, and motivational climate among adolescent football players. Sports (Basel) 7:34. doi: 10.3390/sports7020034, PMID: 30717251 PMC6409893

[ref3] Cox R. H. Martens M. P. Russell W. D. (2003). Measuring anxiety in athletics: the revised competitive state anxiety inventory 2. J. Sport Exerc. Psychol. 25, 519–533. doi: 10.1123/jsep.25.4.519

[ref4] Filippi C. A. Subar A. Ravi S. Haas S. Troller-Renfree S. V. Fox N. A.. (2022). Developmental changes in the association between cognitive control and anxiety. Child Psychiatry Hum. Dev. 53, 599–609. doi: 10.1007/s10578-021-01150-5, PMID: 33738691 PMC9107422

[ref5] Ford J. L. Ildefonso K. Jones M. L. Arvinen-Barrow M. (2017). Sport-related anxiety: current insights. Open Access J. Sports Med. 8, 205–212. doi: 10.2147/OAJSM.S125845 29138604 PMC5667788

[ref6] Hajji J. Elloumi A. (2017). Validation of the Tunisian version of the French version of the competitive state anxiety inventory-2 revised (CSAI-2R), including frequency and direction scales. Int. J. Emerg. Ment. Health 19, 1–7. doi: 10.4172/1522-4821.1000363

[ref7] Hanin Y. L. (2003). Performance related emotional states in sport: a qualitative analysis. Forum qualitative Sozialforschung forum: qualitative. Soc. Res. 4, 1–31. doi: 10.17169/fqs-4.1.747

[ref9] Izard C. E. (2009). Emotion theory and research: highlights, unanswered questions, and emerging issues. Annu. Rev. Psychol. 60, 1–25. doi: 10.1146/annurev.psych.60.110707.163539.2 18729725 PMC2723854

[ref10] Kopp A. Jekauc D. (2018). The influence of emotional intelligence on performance in competitive sports: a Meta-analytical investigation. Sports (Basel). 6:175. doi: 10.3390/sports6040175, PMID: 30551649 PMC6316207

[ref11] Laborde S. Dosseville F. Allen M. S. (2016). Emotional intelligence in sport and exercise: a systematic review. Scand. J. Med. Sci. Sports 26, 862–874. doi: 10.1111/sms.12510 26104015

[ref12] Laborde S. Raab M. Dosseville F. (2013). “Emotions and performance: valuable insights from the sports domain” in Handbook of psychology of emotions (Vol. 1): Recent theoretical perspectives and novel empirical findings. eds. Mohiyeddini C. Eysenck M. Bauer S. (United States: Nova Science Publishers), 25–357.

[ref13] Lane A. M. Beedie C. J. Jones M. V. Uphill M. Devonport T. J. (2012). The BASES expert statement on emotion regulation in sport. J. Sports Sci. 30, 1189–1195. doi: 10.1080/02640414.2012.693621, PMID: 22709410

[ref14] Lane A. M. Thelwell R. C. Lowther J. Devonport T. J. (2009). Emotional intelligence and psychological skills use among athletes. Soc. Behav. Personal. Int. J. 37, 195–201. doi: 10.2224/sbp.2009.37.2.195

[ref15] Lea R. G. Davis S. K. Mahoney B. Qualter P. (2019). Does emotional intelligence buffer the effects of acute stress? Syst. Rev. Front Psychol. 10:810. doi: 10.3389/fpsyg.2019.00810 PMC6478766 31057453

[ref16] Martens R. Burton D. Vealey R. S. Bump L. A. Smith D. E. (1990). “Development and validation of the competitive state anxiety Inventory-2 (CSAI-2)” in Competitive anxiety in sport. eds. Martens R. Vealey R. S. Burton D. (Champaign: Human Kinetics), 117–190.

[ref9001] Martinent G. Ferrand C. Guillet E. Gautheur S. (2010). Validation of the French version of the Competitive State Anxiety Inventory-2 Revised (CSAI-2R) including frequency and direction scales, Psychol. Sport Exerc. 11, 51–57. doi: 10.1016/j.psychsport.2009.05.001

[ref17] Martinent G. Ledos S. Ferrand C. Campo M. Nicolas M. (2015). Athletes’ regulation of emotions experienced during competition: a naturalistic video-assisted study. Sport Exerc. Perform. Psychol. 4, 188–205. doi: 10.1037/spy0000037

[ref18] Mercader-Rubio I. Ángel N. G. Silva S. Brito-Costa S. (2023). Levels of somatic anxiety, cognitive anxiety, and self-efficacy in university athletes from a Spanish public university and their relationship with basic psychological needs. Int. J. Environ. Res. Public Health 20:2415. doi: 10.3390/ijerph20032415, PMID: 36767781 PMC9916372

[ref19] Meyer B. B. Fletcher T. B. (2007). Emotional intelligence: a theoretical overview and implications for research and professional practice in sport psychology. J. Appl. Sport Psychol. 19, 1–15. doi: 10.1080/10413200601102904

[ref21] Parnabas V. A. Mahamood Y. Parnabas J. (2013). The relationship between cognitive and somatic anxiety on performance of student-athletes of Universiti Malaysia Perlis (UNIMAP). Int. J. Human Movement Sports Sci. 1, 61–66. doi: 10.13189/saj.2013.010301

[ref23] Rowland D. L. van Lankveld J. J. D. M. (2019). Anxiety and performance in sex, sport, and stage: identifying common ground. Front. Psychol. 10:1615. doi: 10.3389/fpsyg.2019.01615, PMID: 31379665 PMC6646850

[ref24] Salovey P. E. Sluyter D. J. (1997) Emotional development and emotional intelligence: Educational implications. United States: Basic Books.

[ref25] Sánchez-Álvarez N. Berrios Martos M. P. Extremera N. A. (2020). Meta-analysis of the relationship between emotional intelligence and academic performance in secondary education: a multi-stream comparison. Front. Psychol. 11:1517. doi: 10.3389/fpsyg.2020.01517 32793030 PMC7385306

[ref26] Sanhueza J. A. Zambrano T. Bahamondes-Avila C. Salazar L. A. (2016). Association of Anxiety-Related Polymorphisms with sports performance in Chilean long distance triathletes: a pilot study. J. Sports Sci. Med. 15, 554–561. PMID: 27928199 PMC5131207

[ref27] Schutte N. S. Malouff J. M. Hall L. E. Haggerty D. J. Cooper J. T. Golden C. J.. (1998). Development and validation of a measure of emotional intelligence. Personal. Individ. Differ. 25, 167–177. doi: 10.1016/S0191-8869(98)00001-4

[ref29] Smith D. Bar-Eli M. Essential readings in sport and exercise psychology. United States: DIANE Publishing Inc. (2007).

[ref30] Tamminen K. A. Kim J. Danyluck C. McEwen C. E. CRD W. Wolf S. A. (2021). The effect of self-and interpersonal emotion regulation on athletes’ anxiety and goal achievement in competition. Psychol. Sport Exerc. 57:102034. doi: 10.1016/j.psychsport.2021.102034

[ref31] Tur-Porcar A. Ribeiro-Soriano D. (2020). The role of emotions and motivations in sport organizations. Front. Psychol. 11:842. doi: 10.3389/fpsyg.2020.00842, PMID: 32477215 PMC7233117

[ref32] Tyng C. M. Amin H. U. Saad M. N. M. Malik A. S. (2017). The influences of emotion on learning and memory. Front. Psychol. 8:1454. doi: 10.3389/fpsyg.2017.01454, PMID: 28883804 PMC5573739

[ref33] Üngür G. Karagözoğlu C. (2013). The relationship between emotional intelligence, social physique anxiety and life satisfaction in physical education and sports students. Int. J. Humanit. Soc. Sci. 3, 115–119.

[ref34] Vealey R. S. Campbell J. L. (1988). Achievement goals of adolescent figure skaters: impact on self-confidence, anxiety, and performance. J. Adolesc. Res. 3, 227–243. doi: 10.1177/074355488832009

[ref36] Wagstaff C. R. (2014). Emotion regulation and sport performance. J. Sport Exerc. Psychol. 36, 401–412. doi: 10.1123/jsep.2013-0257 25226609

[ref37] Zoghlami W. Khiari H. Mnedla S. Elloumi A. (2022). Psychometric properties of the Arabic version of the Schutte self-report emotional intelligence test (SSEIT). Int. J. Emerg. Ment. Health 24, 51–56. doi: 10.4172/1522-4821.1000527

